# Comprehensive Analysis of Chyle Leak in Resected Pancreatic Head Cancer: Impact on Clinical, Oncologic, and Nutritional Outcomes

**DOI:** 10.1002/jhbp.12191

**Published:** 2025-08-13

**Authors:** Jae Seung Kwak, Chang Moo Kang, Ho Kyoung Hwang, Sung Hyun Kim, Seung Soo Hong

**Affiliations:** ^1^ Division of Hepatobiliary and Pancreas Surgery, Department of Surgery Yonsei University College of Medicine Seoul Korea; ^2^ Pancreatobiliary Cancer Center Yonsei Cancer Center, Severance Hospital Seoul Korea

**Keywords:** chyle, nutritional status, pancreatic cancer, pancreaticoduodenectomy, postoperative complications

## Abstract

**Background:**

Chyle leak (CL) is a relevant complication of pancreatic surgery, but its incidence, risk factors, clinical and oncologic impacts, and nutritional relevance remain inconsistent and limited.

**Methods:**

We retrospectively reviewed patients who underwent pancreaticoduodenectomy for pancreatic head cancer from 2007 to 2023 at a single institution. The clinical impact of CL was evaluated by prolonged hospital stays and immune‐nutritional status, assessed using the Controlling Nutritional Status (CONUT) score at discharge. Oncologic impact included the administration of adjuvant chemotherapy, the surgery‐to‐chemotherapy interval, overall survival (OS), and recurrence‐free survival (RFS). Predictors of CL were identified through multivariate analyses.

**Results:**

CL occurred in 70 patients (13.8%) and was significantly associated with prolonged hospital stay (OR: 1.947, *p* = 0.045) and poor CONUT score at discharge (> 6; OR: 1.820, *p* = 0.036). CL did not significantly impact oncologic outcomes, including adjuvant chemotherapy (*p* = 0.732), surgery‐to‐chemotherapy interval (*p* = 0.235), 5‐year OS (*p* = 0.978), or 5‐year RFS (*p* = 0.919). Independent predictors of CL included hypertension, lymph node metastasis, delayed gastric emptying, minimally invasive surgery (MIS), and operative time.

**Conclusions:**

CL is associated with prolonged hospital stay and poor nutritional status at discharge, but shows no significant impact on long‐term oncologic outcomes.

## Graphical abstract

Kwak and colleagues conducted a comprehensive analysis of chyle leak in resected pancreatic head cancer using the standardized 2017 ISGPS definition. They confirmed its association with prolonged hospital stay and impaired nutritional status but found no significant oncologic relevance, clarifying previously inconsistent findings due to the lack of a standardized definition.

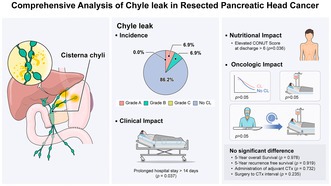

## 1 Introduction

Pancreaticoduodenectomy (PD) is the standard surgical treatment for pancreatic head cancer [1]. Despite advancements in surgical techniques and perioperative care, postoperative complications remain a significant concern. Among these, chyle leak (CL) is a notable postoperative complication of PD, resulting from injury to the cisterna chyli or its tributary lymphatic vessels, which are located near the pancreatic head and neck [2]. Chyle, an emulsion of lymph and triglyceride‐rich fat, plays a critical role in the body's nutritional and immune systems. When a CL occurs, it can lead to malnutrition and an immune‐compromised state, significantly affecting postoperative recovery [3, 4].

With the growing adoption of extended resections to enhance oncologic outcomes, the incidence of CL may rise accordingly [5]. Studies have reported that the incidence of CL ranges from 0.6% to 26.3% and is associated with prolonged hospital stays, higher hospital costs, pancreatic fistula, septic events, and major complications [4, 6, 7, 8, 9, 10, 11]. However, its impact on long‐term outcomes remains unclear [4, 6].

Despite advances in research, the incidence, risk factors, and clinical impacts of CL remain inconsistently reported. In particular, studies on its oncologic significance and association with nutritional status are still limited. One major factor contributing to these discrepancies was the absence of a universally accepted definition of CL, which was only standardized in 2017 by the International Study Group on Pancreatic Surgery (ISGPS). Additionally, variations in study populations and methodologies contribute to inconsistent findings. For instance, many studies include both benign and malignant diseases, even though cancer surgeries typically require more extensive resections due to tumor invasion into surrounding tissues [4, 8, 11]. This difference in surgical extent may affect both the risk and severity of CL. In addition, the inclusion of various surgical procedures—such as PD, distal pancreatectomy, and total pancreatectomy—introduces further heterogeneity [4, 6]. Each procedure presents distinct anatomical and technical challenges that can influence CL development differently, complicating direct comparisons between studies. These inconsistencies and the lack of a comprehensive consensus hinder the development of effective strategies for preventing and managing CL.

To reduce heterogeneity and address research limitations, this study focuses exclusively on patients with pancreatic ductal adenocarcinoma (PDAC) who underwent PD. Using the ISGPS definition of CL, we aim to analyze its incidence, risk factors, clinical and oncologic impact, and nutritional relevance. By applying standardized criteria to a well‐defined patient population, this study seeks to enhance the overall understanding of CL in resected pancreatic head cancer.

## 2 Methods

### 2.1 Patient Selection and Data Acquisition

This study was approved by our Institutional Review Board (IRB No. 4‐2025‐0109). We retrospectively reviewed data from patients who underwent conventional or pylorus‐preserving pancreaticoduodenectomy for pancreatic head cancer, specifically PDAC, between January 2007 and December 2023 at Yonsei University Medical Center, Seoul, Korea. Patients with incomplete medical records or synchronous double primary malignancies in different organs were excluded. Clinicopathological information was collected from institutional medical records for analysis. Patients were categorized into two groups: CL and no CL. The surgical procedure was described in our previous reports [12, 13].

### 2.2 Definition of Variables

We defined CL based on the ISGPS criteria as the presence of milky‐colored fluid from the drain, drain site, or wound on or after postoperative day 3 with a triglyceride level ≥ 110 mg/dL (≥ 1.2 mmol/L) in the fluid. CL was further stratified into Grades A, B, and C according to the ISGPS classification: Grade A represents a clinically irrelevant leak requiring only dietary modification; Grade B involves the need for therapeutic interventions such as total parenteral nutrition, somatostatin analogs, or percutaneous drainage; and Grade C includes severe cases requiring invasive procedures such as reoperation, lymphatic embolization, or intensive care management [5]. Margin status was defined as R0 when the microscopic margin exceeded 1 mm and R1 when it was ≤ 1 mm. Tumor size, nodal status, and metastasis followed the 8th edition of the TNM classification [14]. The Controlling Nutritional Status (CONUT) score, a validated tool for assessing immune‐nutritional status, was calculated based on three parameters: serum albumin level, total lymphocyte count, and total cholesterol level. Each parameter was scored and summed to yield the total CONUT score. Serum albumin levels were scored as follows: ≥ 3.5 g/dL = 0, 3.0–3.49 g/dL = 2, 2.5–2.99 g/dL = 4, and < 2.5 g/dL = 6. Total lymphocyte counts were scored as follows: ≥ 1600/μL = 0, 1200–1599/μL = 1, 800–1199/μL = 2, and < 800/μL = 3. Total cholesterol levels were scored as follows: ≥ 180 mg/dL = 0, 140–179 mg/dL = 1, 100–139 mg/dL = 2, and < 100 mg/dL = 3. The total CONUT score ranged from 0 to 12, with higher scores indicating poorer nutritional status. The preoperative CONUT score, calculated using laboratory results within 30 days before surgery (preferably closest to surgery), was used to evaluate its predictive value for CL. The pre‐discharge CONUT score, used to assess the nutritional impact of CL, was calculated from labs performed a median of 2 days before discharge (interquartile range [IQR]: 1–3). A prolonged hospital stay was defined as more than 14 days, based on the Textbook Outcome definition [15].

The cutoff value for pre‐discharge CONUT score was determined by integrating descriptive and statistical considerations. In the no CL group, the median pre‐discharge CONUT score was 5.5, whereas in the CL group, it was 6. Additionally, receiver operating characteristic (ROC) curve analysis assessing the association between CL and pre‐discharge CONUT score identified potential cutoff values based on the Youden Index, which determines the threshold that maximizes the sum of sensitivity and specificity. Among the candidate values, 4 and 6 exhibited the highest combined values, and given these findings, a cutoff value of 6 was established for this study.

To evaluate the oncologic impact of CL, we analyzed differences between the CL and no CL groups in terms of adjuvant chemotherapy administration, the interval from surgery to adjuvant chemotherapy initiation, overall survival (OS), and recurrence‐free survival (RFS).

### 2.3 Postoperative Nutritional Protocols and Chyle Leak Management

All patients received standardized postoperative nutritional support based on institutional protocols. Patients were kept nil per os on postoperative day (POD) 0. Oral intake was resumed stepwise with a specialized postoperative diet formulated for pancreaticoduodenectomy patients: sips of water (SOW) on POD 1, liquid diet (LD) on POD 2 or 3, and soft diet (SD) as tolerated. Parenteral nutrition (PN) was administered during the early postoperative period at < 1000 kcal/day and tapered as oral intake improved.

In patients diagnosed with chyle leak, a low‐fat or medium‐chain triglyceride (MCT)‐supplemented diet was provided. PN was used when dietary intake was insufficient or restricted. Lipid‐lowering agents such as orlistat were administered upon diagnosis of chyle leak and continued until symptoms improved, typically until discharge. Prophylactic intra‐abdominal drains were routinely placed after surgery. Drain removal was performed at the discretion of the attending surgeon when the output decreased or showed improvement. If necessary, additional percutaneous catheter drainage was performed.

### 2.4 Statistical Analysis

Baseline characteristics were summarized using descriptive statistics. Categorical variables were presented as percentages, and continuous variables as medians with IQRs. Categorical data were analyzed using the chi‐squared or Fisher's exact test, and non‐normally distributed data with the Mann–Whitney *U*‐test. Normality was assessed using the Shapiro–Wilk test. As no variable showed normal distribution, *t*‐tests were not used.

Logistic regression analyses (univariable and multivariable) were used to evaluate associations between CL and hospital stay and between CL and pre‐discharge CONUT score. They were also used to identify independent predictors of CL. Variables with a *p*‐value < 0.20 in the univariable analysis were included in multivariable models using the enter method. Results are presented as odds ratios (ORs) with 95% confidence intervals (CIs). Patients with missing values were excluded prior to conducting univariable and multivariable analyses to ensure consistency in the dataset.

Survival was estimated using Kaplan–Meier analysis, and group differences were compared using the log‐rank test. Univariate and multivariate Cox regression analyses were performed to identify prognostic factors for OS. In these analyses, only significant univariate variables (*p* < 0.05) were included in multivariate Cox analysis to ensure statistical significance and reduce the risk of overfitting.

Statistical significance was defined as a *p* value < 0.05. All statistical analyses were conducted using IBM SPSS Statistics, version 28.0 (IBM Corp., Armonk, NY, USA).

## 3 Results

A total of 523 patients underwent PD between 2007 and 2023. After excluding 15 patients due to incomplete data (*n* = 11, 2.1%) and synchronous double primary malignancies (*n* = 4, 0.8%), a total of 508 patients were enrolled. Among these, 70 patients (13.8%) experienced a CL. Baseline demographic, nutritional, and operative characteristics of the study cohort are summarized in Table 1.

**TABLE 1:** Baseline characteristics.

	Total (*n* = 508)	Chyle leak (*n* = 70)	No chyle leak (*n* = 438)	*p*
Age (years)^a^	64.0 (57.0–70.0)	64.0 (60.0–69.0)	64.0 (57.0–70.0)	0.662^c^
BMI (kg/m^2^)^a^	23.0 (21.2–25.1)	22.9 (21.2–25.0)	23.0 (21.1–25.1)	0.718^c^
Preoperative CONUT score^a^	2 (1–3)	2 (1–3)	2 (1–3)	0.191^c^
Preoperative CA19‐9 (U/mL)				
≤ 37 U/mL	261 (51.4)	35 (50.0)	226 (51.6)	0.804^b^
> 37 U/mL	247 (48.6)	35 (50.0)	212 (48.4)	
Sex				
Male	244 (48.0)	35 (50.0)	209 (47.7)	0.723^b^
Female	264 (52.0)	35 (50.0)	229 (52.3)	
HTN				
Yes	217 (42.7)	41 (58.6)	176 (40.2)	**0.004** ^b^
No	291 (57.3)	29 (41.4)	262 (59.8)	
ASA				
< 3	245 (48.2)	27 (38.6)	218 (49.8)	0.082^b^
≥ 3	263 (51.8)	43 (61.4)	220 (50.2)	
Weight reduction				
Yes	68 (13.4)	16 (22.9)	52 (11.9)	**0.012** ^b^
No	440 (86.6)	54 (77.1)	386 (88.1)	
Neoadjuvant therapy^d^				
Yes	276 (54.3)	43 (61.4)	233 (53.2)	0.199^b^
No	232 (45.7)	27 (38.6)	205 (46.8)	
Operation				
Conventional PD	18 (3.5)	3 (4.3)	15 (3.4)	0.717^b^
PPPD	490 (96.5)	67 (95.7)	423 (96.6)	
Minimally invasive surgery^e^				
Yes	173 (34.1)	15 (21.4)	158 (36.1)	**0.016** ^b^
No	335 (65.9)	55 (78.6)	280 (63.9)	
Surgical approach				
Open	323 (63.6)	55 (78.6)	269 (61.4)	**0.007** ^b^
Laparoscopy	102 (20.1)	5 (7.1)	96 (21.9)	
‐Open conversion	10 (9.8)	0 (0.0)	10 (10.4)	
Robot assisted	83 (16.3)	10 (14.3)	73 (16.7)	
‐Open conversion	2 (2.4)	0 (0.0)	2 (2.7)	
Operative time (hours)^a^	7.3 (5.9–8.8)	6.4 (5.2–7.5)	7.5 (6.1–8.9)	**< 0.001** ^c^
Estimated blood loss (mL)^a^	300 (150–500)	250 (150–450)	300 (150–550)	0.100^c^
Intraoperative transfusion				
Yes	44 (8.7)	2 (2.9)	42 (9.6)	0.068^b^
No	464 (91.3)	68 (97.1)	396 (90.4)	
Postoperative transfusion				
Yes	72 (14.2)	11 (15.7)	61 (13.9)	0.691^b^
No	436 (85.8)	59 (84.3)	377 (86.1)	
Vein resection^f^				
Yes	121 (23.8)	11 (15.7)	110 (25.1)	0.086^b^
No	387 (76.2)	59 (84.3)	328 (74.9)	

*Note:* Bold values indicate statistical significance (*p* < 0.05). Values are presented as number (%) unless otherwise specified.

Abbreviations: ASA, American Society of Anesthesiologists Physical Status Classification; BMI, body mass index; CA19‐9, carbohydrate antigen 19‐9; CONUT, controlling nutritional status; HTN, hypertension; PD, pancreaticoduodenectomy; PPPD, pylorus‐preserving pancreaticoduodenectomy.

^a^ Values are presented as median (IQR).

^b^ Pearson's chi‐squared test.

^c^ Mann–Whitney *U*‐test.

^d^ Neoadjuvant therapy includes patients who received concurrent chemoradiotherapy or chemotherapy alone.

^e^ Patients who underwent open conversion during minimally invasive surgery were counted as “No.”

^f^ It included patients who underwent resection of the SMV, PV, or SV.

### 3.1 Perioperative Outcomes and Clinical Impact of CL

The total cohort had a median tumor size of 2.4 cm (IQR: 1.7–2.8 cm) and a median of 18 retrieved lymph nodes (IQR: 11–26). Regarding nodal status, 49.4% of patients were N0, while 37.0% and 13.6% were N1 and N2, respectively. The margin status indicated that R0 resections were performed in 390 patients (76.8%), while R1 and R2 resections were performed in 118 patients (23.2%). The incidence of postoperative complications included 17.3% for pancreatic fistula (POPF), 4.3% for bile leak, and 11.2% for delayed gastric emptying (DGE).

Patients with CL had a significantly longer hospital stay (> 14 days in 60.0% vs. 47.3%; *p* = 0.048) and a higher proportion of elevated pre‐discharge CONUT scores (> 6 in 42.9% vs. 30.8%; *p* = 0.046) (Table 2). For the cutoff value of 6, the AUC was 0.577 (95% CI: 0.51–0.65), with a sensitivity of 42.9% and a specificity of 69.2%. The timing of pre‐discharge laboratory tests was similar between the CL and No CL groups (median: 2 days, IQR: 1–3, *p* = 0.674), indicating no significant difference between the groups. This suggests that differences in pre‐discharge CONUT scores were not due to discrepancies in the timing of laboratory tests but rather reflected the actual nutritional impact of CL. In the multivariable logistic regression analysis, CL was identified as a significant risk factor for both prolonged hospital stay (> 14 days) (adjusted OR: 1.850, 95% CI: 1.037–3.299, *p* = 0.037) and elevated pre‐discharge CONUT score (> 6) (adjusted OR: 1.820, 95% CI: 1.041–3.185, *p* = 0.036) (Tables 3 and 4).

**TABLE 2:** Postoperative and pathologic outcomes.

	Total (*n* = 508)	Chyle leak (*n* = 70)	No chyle leak (*n* = 438)	*p*
No. retrieved lymph node^a^	18 (11–26)	17 (10–26)	18 (12–26)	0.573^c^
No. positive lymph node^a^	1 (0–2)	1 (0–2)	0 (0–2)	0.107^c^
Tumor size (cm)^a^	2.4 (1.7–2.8)	2.2 (1.7–2.7)	2.4 (1.7–2.8)	0.414^c^
Missing data	5 (1.0)	0 (0.0)	5 (1.1)	
*T* stage^d^				
*T*1	185 (36.8)	25 (35.7)	160 (37.0)	0.386^b^
*T*2	282 (56.1)	39 (55.7)	243 (56.1)	
*T*3	27 (5.4)	3 (4.3)	24 (5.5)	
*T*4	9 (1.8)	3 (4.3)	6 (1.4)	
Missing data	5 (1.0)	0 (0.0)	5 (1.1)	
*N* stage^d^				
*N*0	251 (49.4)	27 (38.6)	224 (51.1)	0.122^b^
*N*1	188 (37.0)	33 (47.1)	155 (35.4)	
*N*2	69 (13.6)	10 (14.3)	59 (13.5)	
Cell differentiation				
Well	71 (14.0)	13 (18.6)	58 (13.2)	0.277^b^
Moderate	327 (64.4)	41 (58.6)	286 (65.3)	
Poor	73 (14.4)	13 (18.6)	60 (13.7)	
Others^e^	8 (1.6)	0 (0.0)	8 (1.8)	
Missing data	29 (5.7)	3 (4.3)	26 (5.9)	
Perineural invasion				
Yes	377 (74.2)	52 (74.3)	325 (74.2)	0.974^b^
No	117 (23.0)	16 (22.9)	101 (23.1)	
Missing	14 (2.8)	2 (2.9)	12 (2.7)	
Lymphovascular invasion				
Yes	172 (33.9)	24 (34.3)	148 (33.8)	0.929^b^
No	322 (63.4)	44 (62.9)	278 (63.5)	
Missing	14 (2.8)	2 (2.9)	12 (2.7)	
*R* status				
*R*0	390 (76.8)	53 (75.7)	337 (76.9)	0.822^b^
*R*1 + *R*2	118 (23.2)	17 (24.3)	101 (23.1)	
POPF				
Yes	88 (17.3)	11 (15.7)	77 (17.6)	0.702^b^
No	420 (82.7)	59 (84.3)	361 (82.4)	
Bile leak				
Yes	22 (4.3)	1 (1.4)	21 (4.8)	0.340^b^
No	486 (95.7)	69 (98.6)	417 (95.2)	
DGE				
Yes	57 (11.2)	2 (2.9)	55 (12.6)	**0.014** ^b^
No	451 (88.8)	68 (97.1)	383 (87.4)	
Wound complication				
Yes	52 (10.2)	9 (12.9)	43 (9.8)	0.436^b^
No	456 (89.8)	61 (87.1)	395 (90.2)	
Other complications^f^				
Yes	177 (34.8)	24 (34.3)	153 (34.9)	0.916^b^
No	331 (65.2)	46 (65.7)	285 (65.1)	
Hospital stay (days)^a^	14 (11–20)	16 (13–21)	14 (11–20)	0.056^c^
Hospital stay				
≤ 14	259 (51.0)	28 (40.0)	231 (52.7)	**0.048** ^b^
> 14	249 (49.0)	42 (60.0)	207 (47.3)	
Adjuvant chemotherapy				
Yes	391 (77.0)	55 (78.6)	336 (76.7)	0.732^b^
No	117 (23.0)	15 (21.4)	102 (23.3)	
Surgery‐to‐chemotherapy interval (days)^a^	55 (46–68)	54 (42–67)	55 (47–68)	0.235^c^
Pre‐discharge CONUT score				
≤ 6	343 (67.5)	40 (57.1)	303 (69.2)	**0.046** ^b^
> 6	165 (32.5)	30 (42.9)	135 (30.8)	

*Note:* Bold values indicate statistical significance (*p* < 0.05). Values are presented as number (%) unless otherwise specified. If missing data are not mentioned, there are no missing data. Missing data were excluded from the analysis Missing data: *T* stage (*n* = 5), cell differentiation (*n* = 29), perineural invasion (*n* = 14), lymphovascular invasion (*n* = 14).

Abbreviations: CONUT, controlling nutritional status; DGE, delayed gastric emptying; No., number; POPF, postoperative pancreatic fistula.

^a^ Values are presented as median (IQR).

^b^ Pearson's chi‐squared test or Fisher's exact test.

^c^ Mann–Whitney *U*‐test.

^d^ *T* stage and *N* stage were classified based on the 8th edition of the American Joint Committee on Cancer (AJCC) staging system.

^e^ The “Others” category includes seven cases of adenosquamous carcinoma and one case of mucinous adenocarcinoma.

^f^ It includes relatively rare complications or extra‐abdominal complications such as pulmonary complications, new‐onset diabetes mellitus, pancreatitis, cholangitis, atrial fibrillation, and sepsis.

**TABLE 3:** Predictors of prolonged hospital stay (> 14 days): Univariate and multivariate analysis.

Variable	Univariate analysis		Multivariate analysis	
	OR (95% CI)	*p*	Adjusted OR (95% CI)	*p*
Age (years)	1.006 (0.988–1.025)	0.500		
Preoperative BMI (kg/m^2^)	1.023 (0.967–1.082)	0.427		
HTN	1.054 (0.742–1.498)	0.769		
DM	0.991 (0.698–1.406)	0.959		
ASA ≥ 3	1.035 (0.731–1.466)	0.847		
Weight loss	0.744 (0.444–1.245)	0.260		
Minimally invasive surgery	0.256 (0.172–0.381)	**< 0.001**	0.172 (0.105–0.282)	**< 0.001**
Operative time (hours)	1.037 (0.956–1.125)	0.387		
Estimated blood loss (mL)	1.000 (1.000–1.000)	0.354		
Intraoperative transfusion	3.035 (1.525–6.040)	**0.002**	2.516 (1.193–5.304)	**0.015**
Postoperative transfusion	1.047 (0.636–1.724)	0.857		
No. retrieved lymph node	0.988 (0.971–1.005)	**0.172**	0.983 (0.964–1.004)	0.106
Lymph node metastasis, positive	1.172 (0.827–1.660)	0.372		
*R* status, *R* ≥ 1	1.313 (0.869–1.985)	**0.196**	1.482 (0.919–2.390)	0.107
Chyle leak	1.674 (1.002–2.798)	**0.049**	1.850 (1.037–3.299)	**0.037**
POPF	3.625 (2.170–6.055)	**< 0.001**	5.390 (2.939–9.883)	**< 0.001**
Bile leak	3.722 (1.352–10.250)	**0.011**	5.063 (1.681–15.248)	**0.004**
DGE	3.300 (1.779–6.123)	**< 0.001**	4.181 (2.077–8.413)	**< 0.001**
Wound complication	2.328 (1.268–4.276)	**0.006**	2.453 (1.237–4.864)	**0.010**
Other complications^a^	1.332 (0.923–1.920)	**0.125**	1.587 (1.028–2.450)	**0.037**
Preoperative CONUT score	1.057 (0.946–1.180)	0.329		

*Note:* Univariate analysis was performed to identify potential factors associated with a prolonged hospital stay (> 14 days). Variables with a *p*‐value < 0.2 in the univariate analysis were included in the multivariate analysis. In the univariate analysis, *p*‐values less than 0.2 were highlighted in bold, while in the multivariate analysis, *p*‐values less than 0.05 were highlighted in bold.

Abbreviations: ASA, American Society of Anesthesiologists Physical Status Classification; BMI, body mass index; CI, confidence interval; CONUT, Controlling Nutritional Status; DGE, delayed gastric emptying; DM, diabetes mellitus; HTN, hypertension; No., number; OR, odds ratio; POPF, postoperative pancreatic fistula.

^a^ It includes relatively rare complications or extra‐abdominal complications such as pulmonary complications, new‐onset diabetes mellitus, pancreatitis, cholangitis, atrial fibrillation, and sepsis.

**TABLE 4:** Predictors of elevated pre‐discharge CONUT score (> 6): Univariate and multivariate analysis.

Variable	Univariate analysis		Multivariate analysis	
	OR (95% CI)	*p*	Adjusted OR (95% CI)	*p*
Age (years)	1.029 (1.008–1.051)	**0.006**	1.018 (0.996–1.041)	0.114
Preoperative BMI (kg/m^2^)	0.917 (0.861–0.977)	**0.007**	0.934 (0.873–0.999)	**0.047**
HTN	1.223 (0.842–1.778)	0.291		
DM	1.239 (0.854–1.800)	0.259		
ASA ≥ 3	0.853 (0.588–1.237)	0.402		
Weight loss	0.993 (0.575–1.714)	0.981		
Minimally invasive surgery	0.746 (0.500–1.113)	**0.151**	0.802 (0.517–1.244)	0.325
Operative time (hours)	1.076 (0.987–1.172)	**0.095**	1.009 (0.905–1.125)	0.875
Estimated blood loss (mL)	1.000 (1.000–1.001)	**0.037**	1.000 (1.000–1.000)	0.577
Intraoperative transfusion	2.484 (1.331–4.634)	**0.004**	1.303 (0.605–2.808)	0.499
Postoperative transfusion	1.296 (0.772–2.175)	0.327		
Vein resection	1.590 (1.041–2.427)	**0.032**	1.644 (1.019–2.652)	**0.042**
Combined organ resection	0.643 (0.252–1.643)	0.357		
Tumor size (cm)^a^	1.269 (1.065–1.511)	**0.008**	1.267 (1.051–1.529)	**0.013**
No. retrieved lymph node	0.986 (0.967–1.004)	**0.128**	0.984 (0.963–1.005)	0.125
Lymph node metastasis, positive	1.220 (0.841–1.770)	0.295		
R status, *R* ≥ 1	1.200 (0.778–1.850)	0.410		
Chyle leak	1.683 (1.006–2.817)	**0.047**	1.820 (1.041–3.185)	**0.036**
POPF	0.946 (0.589–1.578)	0.884		
Bile leak	0.599 (0.217–1.653)	0.323		
DGE	0.717 (0.385–1.334)	0.293		
Wound complication	0.744 (0.391–1.416)	0.368		
Other complications^b^	0.906 (0.612–1.340)	0.621		
Preoperative CONUT score	1.365 (1.208–1.541)	**< 0.001**	1.280 (1.122–1.461)	**< 0.001**

*Note:* Univariate analysis was performed to identify potential factors associated with a pre‐discharge CONUT score (> 6). Variables with a *p*‐value < 0.2 in the univariate analysis were included in the multivariate analysis. In the univariate analysis, *p*‐values less than 0.2 were highlighted in bold, while in the multivariate analysis, *p*‐values less than 0.05 were highlighted in bold.

Abbreviations: ASA, American Society of Anesthesiologists Physical Status Classification; BMI, body mass index; CI, confidence interval; CONUT, Controlling Nutritional Status; DGE, delayed gastric emptying; DM, diabetes mellitus; HTN, hypertension; No., number; OR, odds ratio; POPF, postoperative pancreatic fistula.

^a^ This variable contains five missing values. Missing data were excluded from the analysis.

^b^ It includes relatively rare complications or extra‐abdominal complications such as pulmonary complications, new‐onset diabetes mellitus, pancreatitis, cholangitis, atrial fibrillation, and sepsis.

### 3.2 Predicting Factors for CL

Univariate and multivariate analyses were conducted to identify significant predictors of CL (Table 5). In the multivariate analysis, HTN (adjusted OR: 2.032, 95% CI: 1.148–3.597, *p =* 0.015) and lymph node metastasis (adjusted OR: 1.872, 95% CI: 1.067–3.287, *p =* 0.029) were significantly associated with an increased risk of CL. In contrast, minimally invasive surgery (adjusted OR: 0.491, 95% CI: 0.256–0.942, *p =* 0.032), longer operative time (adjusted OR: 0.741, 95% CI: 0.623–0.882, *p =* 0.001), and DGE (adjusted OR: 0.150, 95% CI: 0.034–0.654, *p =* 0.012) were associated with a reduced risk.

**TABLE 5:** Predicting factor of CL.

Variable	Univariate analysis		Multivariate analysis	
	OR (95% CI)	*p*	Adjusted OR (95% CI)	*p*
Age (years)	1.007 (0.980–1.034)	0.627		
Sex, female	0.913 (0.551–1.512)	0.723		
Preoperative BMI (kg/m^2^)	0.983 (0.906–1.067)	0.678		
HTN	2.105 (1.261–3.514)	**0.004**	2.032 (1.148–3.597)	**0.015**
DM	1.594 (0.960–2.646)	**0.071**	1.433 (0.782–2.625)	0.245
ASA ≥ 3	1.578 (0.942–2.645)	**0.083**	1.324 (0.706–2.485)	0.381
Cancer history	1.705 (0.809–3.595)	**0.161**	1.526 (0.679–3.429)	0.306
Previous abdominal surgery	1.131 (0.563–2.271)	0.730		
Preoperative jaundice	0.668 (0.364–1.226)	**0.192**	0.733 (0.383–1.400)	0.346
Weight loss	2.199 (1.173–4.124)	**0.014**	1.781 (0.900–3.526)	0.097
Minimally invasive surgery	0.483 (0.264–0.884)	**0.018**	0.491 (0.256–0.942)	**0.032**
Operative time (hours)	0.737 (0.637–0.852)	**< 0.001**	0.741 (0.623–0.882)	**0.001**
Estimated blood loss (mL)	0.999 (0.999–1.000)	**0.171**	1.000 (0.999–1.001)	0.818
Vein resection	0.556 (0.282–1.096)	**0.090**	0.699 (0.329–1.486)	0.352
Combined organ resection	1.203 (0.400–3.617)	0.741		
No. retrieved lymph node	0.995 (0.970–1.020)	0.671		
*T* stage, *T* stage ≥ 2	1.055 (0.560–1.605)	0.842		
Lymph node metastasis, positive	1.667 (0.995–2.794)	**0.052**	1.872 (1.067–3.287)	**0.029**
POPF	0.874 (0.439–1.741)	0.702		
Bile leak	0.288 (0.038–2.174)	0.227		
Delayed gastric emptying	0.205 (0.049–0.860)	**0.030**	0.150 (0.034–0.654)	**0.012**
Preop CONUT score	1.101 (0.945–1.282)	0.216		

*Note:* Univariate analysis was performed to identify potential factors associated with a chyle leak. Variables with a *p*‐value < 0.2 in the univariate analysis were included in the multivariate analysis. In the univariate analysis, *p*‐values less than 0.2 were highlighted in bold, while in the multivariate analysis, *p*‐values less than 0.05 were highlighted in bold.

Abbreviations: ASA, American Society of Anesthesiologists Physical Status Classification; BMI, body mass index; CI, confidence interval; CONUT, controlling nutritional status; DM, diabetes mellitus; HTN, hypertension; No., number; OR, odds ratio; POPF, postoperative pancreatic fistula.

### 3.3 Oncologic Impact of CL

**FIGURE 1:**
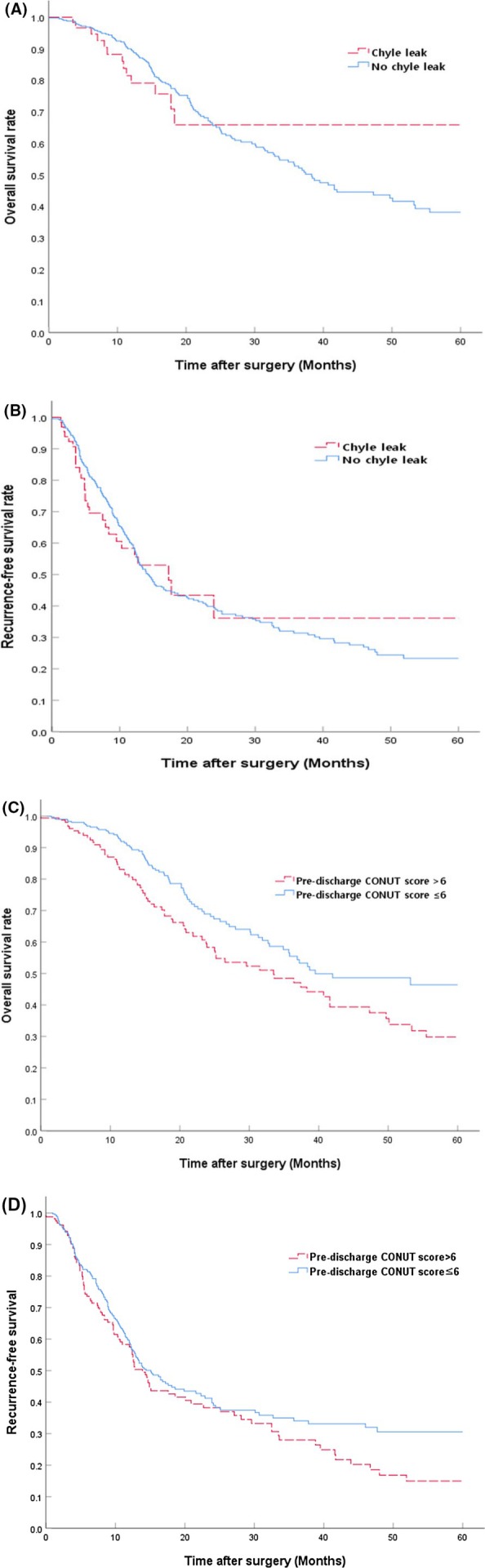
Kaplan–Meier survival analyses were performed to compare overall survival (OS) and recurrence‐free survival (RFS) rates based on two factors: Chyle leak versus no chyle leak and pre‐discharge CONUT score > 6 versus pre‐discharge CONUT score ≤ 6. (A) The 5‐year OS rate was 65.8% in the chyle leak group and 38.1% in the no chyle leak group (*p* = 0.978). (B) The 5‐year RFS rate was 36.1% in the chyle leak group and 23.3% in the no chyle leak group (*p* = 0.919). (C) The 5‐year OS rate was significantly lower in patients with a pre‐discharge CONUT score > 6 (29.8%) compared to those with a CONUT score ≤ 6 (46.4%) (*p* = 0.009). (D) The 5‐year RFS rate was 15.0% in patients with a CONUT score > 6 and 30.5% in those with a CONUT score ≤ 6, with no statistically significant difference between the groups (*p* = 0.118).

There was no statistically significant difference in the administration of adjuvant chemotherapy between the CL group and the no CL group (78.6% vs. 76.7%; *p* = 0.732). Similarly, the surgery‐to‐chemotherapy interval was comparable between the two groups (median 54 days [42–67] vs. 55 days [47–68]; *p* = 0.235) (Table 2). Kaplan–Meier survival analyses showed no significant differences between the CL group and the no CL group in 5‐year OS (65.8% vs. 38.1%, *p* = 0.978) or 5‐year RFS (36.1% vs. 23.3%, *p* = 0.919) (Figure 1A,B). Moreover, Cox proportional hazards regression analysis confirmed that CL was not an independent prognostic factor for OS (Table 6). These findings collectively suggest that CL does not significantly impact long‐term oncologic outcomes in patients with resected pancreatic head cancer.

**TABLE 6:** Prognostic factors for overall survival.

Variable	Univariate analysis		Multivariate analysis	
	OR (95% CI)	*p*	Adjusted OR (95% CI)	*p*
Age (years)	1.017 (0.999–1.035)	0.059		
Sex, female	1.021 (0.741–1.406)	0.901		
Preoperative BMI (kg/m^2^)	0.961 (0.913–1.012)	0.134		
HTN	0.924 (0.667–1.281)	0.636		
DM	1.259 (0.913–1.735)	0.160		
ASA ≥ 3	1.146 (0.831–1.579)	0.406		
Cancer history	0.866 (0.480–1.562)	0.633		
Preoperative jaundice	0.922 (0.649–1.309)	0.649		
Weight loss	1.128 (0.704–1.807)	0.617		
Preoperative CA19‐9 > 37 U/mL	2.216 (1.582–3.104)	**< 0.001**	1.884 (1.270–2.796)	**0.002**
Minimally invasive surgery	0.694 (0.446–1.078)	0.104		
Intraoperative transfusion	2.177 (1.435–3.303)	**< 0.001**	1.978 (1.229–3.184)	**0.005**
Postoperative transfusion	2.261 (1.606–3.182)	**< 0.001**	1.904 (1.325–2.735)	**< 0.001**
Vein resection	1.382 (0.984–1.942)	0.062		
Combined organ resection	1.659 (0.897–3.068)	0.106		
*T* stage^a^		**0.001**		
*T*1	Reference			
*T*2	1.880 (1.294–2.730)		0.968 (0.622–1.505)	0.884
*T*3	2.799 (1.519–5.155)		1.321 (0.665–2.624)	0.427
*T*4	1.155 (0.158–8.433)		0.953 (0.125–7.261)	0.963
*N* stage		**< 0.001**		
*N*0	Reference		Reference	
*N*1	2.151 (1.486–3.115)		1.688 (1.090–2.615)	**0.019**
*N*2	4.114 (2.667–6.346)		2.645 (1.528–4.578)	**0.001**
Retrieved lymph node	1.000 (0.984–1.017)	0.961		
*R* status, *R* ≥ 1	1.315 (0.920–1.880)	0.133		
Cell differentiation^a^		**0.016**		
Well	Reference		Reference	
Moderate	1.812 (1.018–3.225)		1.435 (0.777–2.651)	0.248
Poor	2.679 (1.381–5.199)		3.032 (1.478–6.221)	**0.002**
Others	0.507 (0.066–3.881)		0.540 (0.067–4.332)	0.562
Lymphovascualr invasion^a^	2.624 (1.895–3.633)	**< 0.001**	2.134 (1.429–3.187)	**< 0.001**
Perineural invasion^a^	1.887 (1.217–2.924)	**0.005**	1.132 (0.701–1.829)	0.612
Chyle leak	1.008 (0.569–1.786)	0.978		
POPF	0.832 (0.554–1.249)	0.375		
Bile leak	0.760 (0.335–1.720)	0.510		
DGE	1.038 (0.665–1.619)	0.871		
Wound complication	0.735 (0.407–1.326)	0.306		
Other complications^b^	1.329 (0.949–1.861)	0.098		
Neoadjuvant therapy	0.692 (0.500–0.957)	**0.026**	1.279 (0.884–1.849)	0.192
Adjuvant chemotherapy	0.649 (0.432–0.973)	**0.037**	0.520 (0.336–0.804)	**0.003**
Surgery to adjuvant chemotherapy interval (days)	1.004 (0.999–1.008)	0.085		
Surgery to adjuvant chemotherapy interval > 12 weeks	1.525 (0.900–2.582)	0.117		
Preoperative CONUT score	1.153 (1.053–1.263)	**0.002**	1.115 (1.011–1.231)	**0.029**
Pre‐discharge CONUT score > 6	1.532 (1.110–2.114)	**0.009**	1.310 (0.911–1.886)	0.146

*Note:* To identify prognostic factors for overall survival (OS), variables with a *p*‐value less than 0.05 in the univariate analysis were included in the multivariate analysis to determine independent prognostic factors. Bold values indicate statistical significance (*p* < 0.05).

Abbreviations: ASA, American Society of Anesthesiologists Physical Status Classification; BMI, body mass index; CA19‐9, carbohydrate antigen 19‐9; CI, confidence interval; CONUT, Controlling Nutritional Status; DGE, delayed gastric emptying; DM, diabetes mellitus; HTN, hypertension; OR, odds ratio; POPF, postoperative pancreatic fistula.

^a^ These factors include missing data, which are detailed in Table 2. The analyses were performed after excluding missing data.

^b^ It includes relatively rare complications or extra‐abdominal complications such as pulmonary complications, new‐onset diabetes mellitus, pancreatitis, cholangitis, atrial fibrillation, and sepsis.

We performed an additional analysis stratified by CL severity based on the ISGPS classification. In our cohort, 35 patients (6.9%) were classified as grade A, 35 (6.9%) as grade B, and none as grade C. Therefore, we compared oncologic outcomes between patients with grade B CL and those with either no CL or grade A CL. The rate of adjuvant chemotherapy administration was similar between the two groups (77.1% in the grade B CL group vs. 77.0% in the no or grade A CL group; *p* = 0.980). Among patients who received adjuvant treatment (*n* = 391), the median interval from surgery to chemotherapy was 49 days (IQR: 40–68) in the grade B group and 55 days (IQR 47–68) in the no or grade A group, with no statistically significant difference (*p* = 0.146) (Table S1). In Kaplan–Meier survival analyses, there were no significant differences in 5‐year overall survival (Figure S1A, log‐rank *p* = 0.677) or 5‐year recurrence‐free survival (Figure S1B, log‐rank *p* = 0.576) between the two groups. Furthermore, in the univariate Cox proportional hazards analysis, grade B CL was not identified as a prognostic factor for overall survival (HR: 0.840; 95% CI: 0.371–1.904; *p* = 0.677). Accordingly, it was not included in the multivariate model due to lack of statistical significance in the univariate analysis.

## 4 Discussion

CL occurred in 13.8% of patients and was associated with prolonged hospitalization and poor nutritional status at discharge, but not with oncologic outcomes. Independent risk factors for CL included HTN and lymph node metastasis. Conversely, minimally invasive surgery, prolonged operative time, and DGE were associated with a reduced risk of CL.

The 13.8% CL incidence observed in this study falls within the previously reported range of 0.6%–26.3% [4, 6, 7, 8, 9, 10, 11]. This slightly higher rate may be attributed to our exclusive inclusion of malignant tumors located in the pancreatic head, which required PD. Malignancy and the proximity of the pancreatic head to major lymphatic structures likely increased CL risk. Strobel et al. [4] identified resection for malignancy as an independent risk factor for CL after pancreatic surgery. Additionally, a systematic review reported that PD is associated with a higher incidence of CL compared to other pancreatic surgeries, such as total pancreatectomy and distal pancreatectomy [16]. A recent study using the ISGPS definition and including a similar cohort reported a similar incidence of CL at 12% [11].

Patients with CL exhibited prolonged hospital stays and elevated CONUT scores at discharge. Previous studies have reported an association between CL and prolonged hospitalization [4, 10]. However, we could not identify any previous reports that have specifically focused on postoperative immune‐nutritional status in relation to CL. In this study, we utilized the CONUT score for evaluating immune‐nutritional status, both preoperatively and at discharge. Among several indices, the CONUT score was selected for two main reasons. First, it includes serum albumin, lymphocyte count, and total cholesterol—parameters that align with chyle's major components: albumin, lymphocytes, and triglycerides [17]. While total cholesterol is not directly linked to triglycerides, it reflects lipid metabolism. Second, it's proven significance as a prognostic factor for long‐term survival in patients with PDAC [18]. This made it suitable for evaluating the nutritional impact of CL and its potential indirect influence on survival, as CL was a significant predictor of elevated pre‐discharge CONUT scores.

The preoperative CONUT score showed no association with the occurrence of CL, consistent with previous findings [19]. CONUT scores around postoperative day 3 were sometimes more favorable than preoperative scores, likely due to confounders like postoperative albumin administration, limiting interpretability. Around the time of discharge, we observed a significantly higher proportion of patients with a pre‐discharge CONUT score > 6 in the CL group (42.9% vs. 30.8%, *p* = 0.046). This compromised immune‐nutritional status may impair postoperative recovery [20] and potentially impact oncologic outcomes [21]. In this study, patients with a pre‐discharge CONUT score > 6 had a significantly longer interval from surgery to chemotherapy (median: 57 days [48–78] vs. 54 days [45–65]; *p* = 0.020, Table S2) and lower 5‐year OS (Figure 1C) than those with scores ≤ 6. However, there were no significant differences in the administration rate of adjuvant chemotherapy (72.7% vs. 79.0%; *p* = 0.115, Table S2) or 5‐year RFS (Figure 1D) between the two groups. Although CL itself did not directly reduce the administration of adjuvant chemotherapy or prolong the surgery‐to‐chemotherapy interval—further logistic regression analysis revealed that CL was not an independent risk factor for the non‐administration of adjuvant chemotherapy (*p* = 0.732) or the prolongation of the surgery‐to‐chemotherapy interval (*p* = 0.268)—it may have the potential to indirectly influence the interval by worsening immune‐nutritional status, which warrants further investigation in larger datasets. Although the optimal timing of chemotherapy remains debated [22, 23], our findings underscore the need for proactive nutritional support, such as early parenteral nutrition or lipid‐adjusted diets, to mitigate the impact of CL on immune‐nutritional status and prevent its potential negative effects on oncologic outcomes.

Lymph node metastasis and HTN were identified as independent risk factors for CL, whereas MIS, longer operative time, and DGE were independent protective factors against CL. Previous studies reported various predictors, including open surgery, distal pancreatectomy, pancreatic carcinoma, diabetes mellitus, operation time, number of harvested lymph nodes, para‐aortic lymph node sampling, manipulating the para‐aortic area, manipulating the SMA root area, vascular resection, focal chronic pancreatitis, retroperitoneal invasion, and late enteral feeding time [4, 6, 8, 24, 25]. Augustinus et al. [8] reported open surgery as a risk factor for CL, supporting our finding of MIS as a protective factor against CL. The role of operative time remains controversial. Kim et al. [26] identified shorter operative time as a risk factor for CL and suggested that reduced tissue inflammation may preserve lymphatic flow, increasing CL risk if lymphatic vessels are injured—while also contributing to shorter operative duration. In contrast, Strobel et al. [4] reported that an operative time of ≥ 180 min was a risk factor for CL. The rationale behind our finding that longer operative time serves as a protective factor against CL remains unclear and complex. Since MIS was identified as an independent protective factor against CL in our study and is generally associated with a longer operative time (median operative time: open surgery, 7.1 h vs. MIS, 7.6 h), the observed protective effect of longer operative time on CL may have been influenced by the difference in surgical approaches rather than the operative time itself. However, this interpretation requires further investigation. Lymph node metastasis was a risk factor in our study, but not the number of positive or harvested lymph nodes. In contrast, Shyr et al. [11] identified the number of involved lymph nodes as a risk factor for CL, but multivariate analysis was not performed. Assumpcao et al. [6] identified the number of harvested lymph nodes as a risk factor. These discrepancies may result from differences in study populations, surgical techniques, and statistical methodologies. Our study exclusively included patients with malignant tumors in the pancreatic head who underwent PD, whereas previous studies may have included heterogeneous cohorts with varying tumor sites and surgical procedures. Additionally, the absence of multivariate logistic regression analysis in some studies limits their ability to determine independent risk factors. Variations in CL definitions and diagnostic criteria may also contribute to inconsistent findings across studies.

To our knowledge, DGE and HTN have not been previously reported as predictors of CL. As parenteral nutrition and dietary restriction are commonly used in managing grade B or C CL, DGE may appear protective because its management often involves strategies such as delayed progression to solid food, nasogastric tube insertion, or total parenteral nutrition, depending on severity [6, 27]. Research on the effects of HTN on the lymphatic system is limited. Mukohda et al. [28] reported that increased blood pressure can cause lymphatic endothelial dysfunction via oxidative stress, leading to compromised integrity of lymphatic vessels. This may increase vulnerability to surgical injury and result in a higher incidence of CL [28]. Additionally, HTN is associated with delayed wound healing and increased risk of postoperative complications, which can further exacerbate the likelihood of developing CL [29, 30]. The impaired healing process may prolong lymphatic leakage and complicate management. Because studies on the relationship between HTN and CL are limited, further research is warranted to establish a definitive causal relationship and to develop targeted interventions for patients with HTN undergoing surgeries with a high risk of CL.

This study confirmed that CL is not a prognostic factor for OS. Similarly, a previous study also demonstrated no significant impact of chyle leak on long‐term outcomes, except in patients who underwent palliative procedures and failed to resolve the leak within 14 days of treatment, which was associated with worse survival [4]. While a pre‐discharge CONUT score > 6 was identified as a potential prognostic factor for OS in univariate analysis (*p* = 0.009), it did not remain significant in multivariate analysis (*p* = 0.146). Therefore, although CL was a risk factor for a pre‐discharge CONUT score > 6, it did not indirectly affect OS. Similarly, while CL was a risk factor for a pre‐discharge CONUT score > 6, and a pre‐discharge CONUT score > 6 significantly prolonged the surgery‐to‐chemotherapy interval, this delay—often debated as a prognostic factor for OS [22, 23]—was not identified as a prognostic factor for OS in our study. Thus, CL did not indirectly affect OS through this mechanism either.

This study has several limitations to consider. First, its retrospective design and single‐center dataset may limit generalizability. Although the sample size was large, selection bias is possible due to the exclusion of patients with incomplete records. Second, although we defined CL based on the ISGPS criteria, grading accuracy may have been limited due to the retrospective nature of the study. Therefore, we focused our analysis on the presence or absence of chyle leak rather than its severity grade. Further prospective studies are needed to validate our findings using more accurate grading of CL. Third, while we identified independent prognostic factors for OS, interactions among variables such as tumor biology, surgical complexity, and nutritional recovery remain complex. Prospective multicenter studies with more comprehensive data are needed to validate these findings and assess causality. Lastly, assessing immune‐nutritional status in the immediate postoperative period may be subject to significant variability. However, in this study, the pre‐discharge CONUT score was assessed just before discharge, when patients were generally stable and no longer receiving interventions like transfusions or albumin supplementation that could affect nutritional values. This makes the pre‐discharge assessment a valuable parameter for study. As research on postoperative nutritional status at discharge is extremely limited, further studies in this area are warranted. A major strength of this study is its exclusive focus on patients with pancreatic head cancer undergoing PD, thereby minimizing population heterogeneity. It also ensures that consistency by utilizing the ISGPS definition and comprehensively analyzes clinical, oncologic, and nutritional outcomes to address gaps in existing research.

In conclusion, CL is associated with prolonged hospital stays and impaired immune‐nutritional status at discharge, but does not affect long‐term oncologic outcomes. In addition, CL was identified as a risk factor for a pre‐discharge CONUT score > 6, which increased the surgery‐to‐chemotherapy interval, indicating that CL may potentially have a limited indirect impact on this oncologic outcome. These findings suggest that while CL has clinical significance, its impact on oncologic outcomes remains limited in patients with resected pancreatic head cancer.

## Conflicts of Interest

The authors declare no conflicts of interest.

## Supporting information


**Figure S1.** Subgroup analysis of survival outcomes according to chyle leak severity. (A) Kaplan–Meier analysis of overall survival comparing patients with grade B CL and those with no or grade A CL (log‐rank *p* = 0.677). (B) Kaplan–Meier analysis of recurrence‐free survival comparing the same two groups (log‐rank *p* = 0.576).

**Table S1.** Comparison of adjuvant chemotherapy and surgery‐to‐chemotherapy interval according to chyle leak severity (Grade B vs. No or Grade A CL).

**Table S2.** Association of pre‐discharge CONUT score with adjuvant chemotherapy and surgery‐to‐chemotherapy interval.

## Data Availability

The data that support the findings of this study are available on request from the corresponding author. The data are not publicly available due to privacy or ethical restrictions.
