# Supplementary figures and images for: Comprehensive Analysis of Chyle Leak in Resected Pancreatic Head Cancer: Impact on Clinical, Oncologic, and Nutritional Outcomes
Source: J Hepatobiliary Pancreat Sci. 2025 Aug 13;32(10):787–800. doi: 10.1002/jhbp.12191 (PMC12559876; doi:10.1002/jhbp.12191)

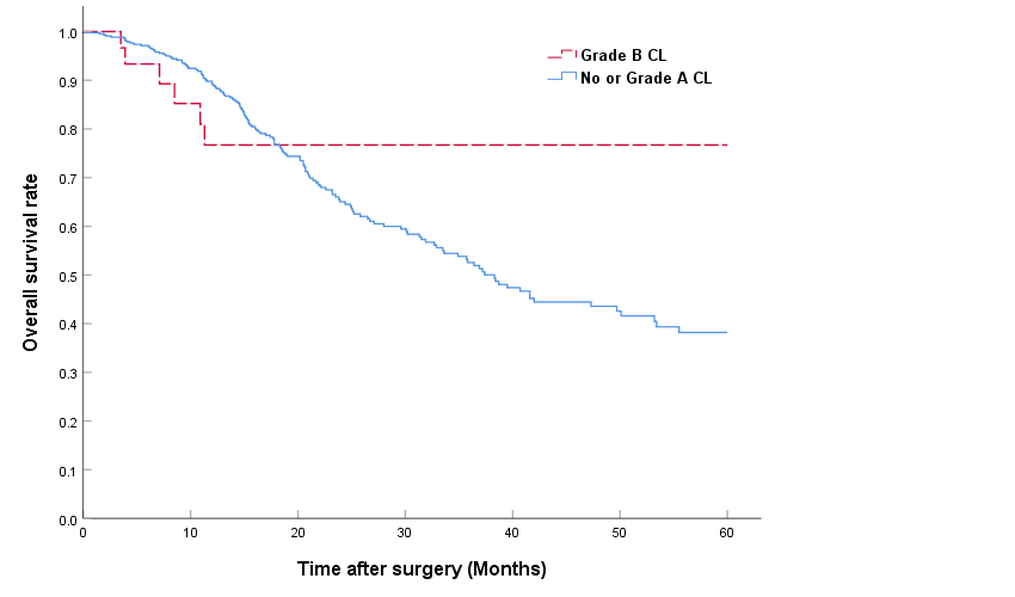

Supplement: Supplementary file 1 — Figure S1. Subgroup analysis of survival outcomes according to chyle leak severity. (A) Kaplan–Meier analysis of overall survival comparing patients with grade B CL and those with no or grade A CL (log‐rank p = 0.677). (B) Kaplan–Meier analysis of recurrence‐free survival comparing the same two groups (log‐rank p = 0.576). [file JHBP-32-787-s003.zip › jhbp12191-sup-0002-FigureS1@Supplementary figure 1A.tif]

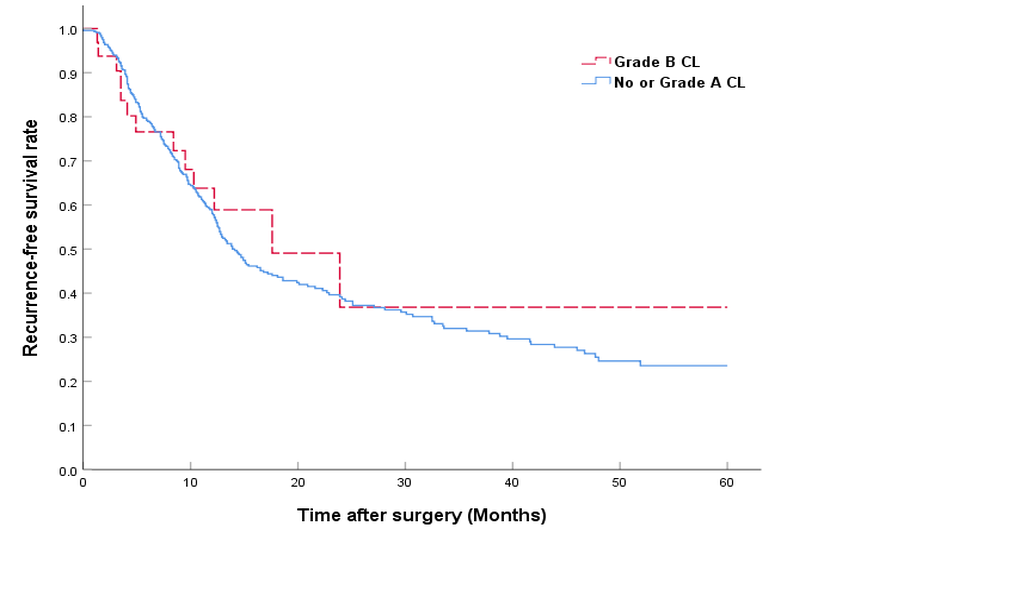

Supplement: Supplementary file 1 — Figure S1. Subgroup analysis of survival outcomes according to chyle leak severity. (A) Kaplan–Meier analysis of overall survival comparing patients with grade B CL and those with no or grade A CL (log‐rank p = 0.677). (B) Kaplan–Meier analysis of recurrence‐free survival comparing the same two groups (log‐rank p = 0.576). [file JHBP-32-787-s003.zip › jhbp12191-sup-0003-FigureS1@Supplementary figure 1B.tif]
