# Supplementary material for: Comprehensive Analysis of Chyle Leak in Resected Pancreatic Head Cancer: Impact on Clinical, Oncologic, and Nutritional Outcomes
Source: J Hepatobiliary Pancreat Sci. 2025 Aug 13;32(10):787–800. doi: 10.1002/jhbp.12191 (PMC12559876; doi:10.1002/jhbp.12191)
Supplement: Supplementary file 2 — Table S1. Comparison of adjuvant chemotherapy and surgery‐to‐chemotherapy interval according to chyle leak severity (Grade B vs. No or Grade A CL). [file JHBP-32-787-s002.docx]

Supplementary Table 1) Comparison of Adjuvant Chemotherapy and Surgery-to-Chemotherapy Interval According to Chyle Leak Severity (Grade B vs. No or Grade A CL)

|  | Total  (n=508) | Grade B CL  (n=35) | No or Grade A CL  (n=473) | *p-*value |
| --- | --- | --- | --- | --- |
| Adjuvant chemotherapy |  |  |  | 0.980 |
| Yes | 391 (77.0) | 27 (77.1) | 364 (77.0) |  |
| No | 117 (23.0) | 8 (22.9) | 109 (23.0) |  |
| Surgery-to-chemotherapy interval*^1)^ (days) | 55 (46-68) | 49 (40-68) | 55 (47-68) | 0.146 |

Values are presented as number (%) unless otherwise specified; * values are presented as median (IQR).

1) It was analyzed among the 391 patients who received adjuvant chemotherapy (Grade B CL group: 27 patients; No or Grade A CL group: 364 patients)
