# Supplementary material for: Comprehensive Analysis of Chyle Leak in Resected Pancreatic Head Cancer: Impact on Clinical, Oncologic, and Nutritional Outcomes
Source: J Hepatobiliary Pancreat Sci. 2025 Aug 13;32(10):787–800. doi: 10.1002/jhbp.12191 (PMC12559876; doi:10.1002/jhbp.12191)
Supplement: Supplementary file 3 — Table S2. Association of pre‐discharge CONUT score with adjuvant chemotherapy and surgery‐to‐chemotherapy interval. [file JHBP-32-787-s001.docx]

Supplementary Table 2) Association of Pre-discharge CONUT Score with Adjuvant Chemotherapy and Surgery-to-chemotherapy Interval

|  | Total  (n=508) | Pre-discharge CONUT score >6  (n=165) | Pre-discharge CONUT score ≤6  (n=343) | *p* |
| --- | --- | --- | --- | --- |
| Adjuvant chemotherapy |  |  |  | 0.115 |
| Yes | 391(77.0) | 120(72.7) | 271(79.0) |  |
| No | 117(23.0) | 45(27.3) | 72(21.0) |  |
| Surgery-to-chemotherapy interval*^1)^ (days) | 55(46-68) | 57(48-78) | 54(45-65) | **0.020** |

Values are presented as number (%) unless otherwise specified; * values are presented as median (IQR).

1) It was analyzed among the 391 patients who received adjuvant chemotherapy (Pre-discharge CONUT score>6 group: 120 patients; Pre-discharge CONUT score ≤6 group: 271 patients)
